# Effect of Hepatitis C Drugs on Blood Coagulability in Patients on Warfarin Using the Medical Information Database Network (MID-NET^®^) in Japan

**DOI:** 10.1007/s43441-020-00247-8

**Published:** 2021-01-03

**Authors:** Sono Sawada, Takashi Ando, Mai Hirano, Noriyuki Komiyama, Toyotaka Iguchi, Yukio Oniyama, Chieko Ishiguro, Yoshiaki Uyama

**Affiliations:** 1 Office of Medical Informatics and Epidemiology, Pharmaceuticals and Medical Devices Agency, Shin-Kasumigaseki Building, 3-3-2 Kasumigaseki, Chiyodaku, Tokyo, 100-0013 Japan; 2 Office of Pharmacovigilance II, Pharmaceuticals and Medical Devices Agency, Tokyo, Japan; 3 Office of Pharmacovigilance I, Pharmaceuticals and Medical Devices Agency, Tokyo, Japan

## Abstract

**Background:**

Previous studies suggested that direct-acting antivirals (DAAs) against hepatitis C increased the blood coagulability of patients on warfarin. This study aims to descriptively investigate the effects of DAAs on the blood coagulability and liver function of patients on warfarin in Japan.

**Methods:**

The Medical Information Database Network (MID-NET^®^) was used as data source. Fluctuations of blood coagulability and liver function were examined before and after DAA treatment in patients who were prescribed both DAAs and warfarin at least once during the study period from January 1, 2010, to December 31, 2017.

**Results:**

For the 16 eligible patients, the mean values of both PT-INR and WSI (warfarin sensitivity index) defined as the value obtained by dividing the PT-INR by the warfarin daily dose slightly decreased at the date of completion of the DAA treatment in comparison with those at the date of initiation and subsequently increased at 12 weeks after treatment completion. In contrast, the warfarin daily dose increased at the date of completion of the DAA treatment, followed by a decrease at 12 weeks after its completion. Several laboratory tests related to the liver function also revealed a similar decrease at the end of the DAA treatment.

**Conclusion:**

The analysis of MID-NET^®^ data provides useful information on drug safety assessment of real-world patients. The results of this study imply that fluctuation of the liver function test results may relate to the fluctuation of blood coagulability in patients on both DAA and warfarin. This study contributes to a deeper understanding of the usefulness and limitations of real-world data in MID-NET^®^ for regulatory purposes.

**Supplementary information:**

The online version of this article (10.1007/s43441-020-00247-8) contains supplementary material, which is available to authorized users.

## Background

According to the 2019 Japanese hepatitis C treatment guidelines, the number of hepatitis C patients in Japan was approximately 1 to 1.5 million [1]; particularly, the highest proportion of hepatitis C virus (HCV) carriers among the entire population was observed in the cohort aged over 40 in 2000 [2], while corresponding to the cohort aged over 60 in 2020. Telaprevir has been the first direct-acting antiviral against HCV to be approved in Japan in 2011, followed by the approval of other types of DAAs including interferon-free DAAs from 2014 [3]. Interferon-free DAAs showed a higher effectiveness in achieving sustained virological response (SVR) rates (90–95%) in comparison with interferon (SVR rates: 42–52%) [4, 5].

Previous studies suggested that DAAs increase the blood coagulability in patients on the anticoagulant warfarin [6–8]. In particular, three possible reasons for the fluctuation of blood coagulability during DAA administration were proposed: (1) improved liver function derived from the elimination of HCV; (2) a certain drug-drug interaction between warfarin and the DAA or ritonavir co-administered with the DAA; (3) primarily, co-administration of ribavirin with the DAA.

In September 2016, the European DAA package inserts were revised to add a precaution about blood coagulation in patients treated with vitamin K antagonists, based on a recommendation from the Pharmacovigilance Risk Assessment Committee of the European Medicine Agency (EMA) [9]. Similar revisions were also made in the US after November 2017 [10]. The Pharmaceuticals and Medical Devices Agency (PMDA) continuously monitors the safety of DAAs in relation to blood coagulability in patients on warfarin by assessing spontaneous adverse event reports and collecting the information on safety measures taken by foreign regulatory agencies such as EMA and US-FDA. In order to further investigate the effects of DAAs on blood coagulability in patients on warfarin in Japan, the PMDA decided to conduct a study by utilizing the new Japanese medical information database network (MID-NET^®^) that was officially launched in April 2018 [11]. In this study, fluctuations of the liver function were also examined for achieving a better understanding of the relationship between liver function and blood coagulability. This study describes the MID-NET^®^ investigation and PMDA consideration about the effects of DAAs on blood coagulability in patients on warfarin in Japan.

## Methods

### Data Source

In this study, data from MID-NET^®^, which is known to be a reliable and valuable database in Japan [11, 12], were used, because MID-NET^®^ stores electronic medical records, administrative claim data and diagnosis procedure combination (DPC) data of about 5.1 million patients in cooperation with 10 healthcare organizations including 23 university hospitals or regional core hospitals, and quantitative and longitudinal data of relevant laboratory tests such as prothrombin time-international normalized ratio (PT-INR), aspartate aminotransferase (AST), alanine aminotransferase (ALT), alkaline phosphatase (ALP), γ-glutamyl transpeptidase (γ-GTP), index of hepatic fibrosis (FIB-4 index), platelet count, and hepatitis C viral load were available for analysis. The study period was from January 1, 2010, to December 31, 2017, and involved 22 hospitals, which corresponded to the latest and maximal data available at the time of data extraction.

### Cohort

**Figure 1.**
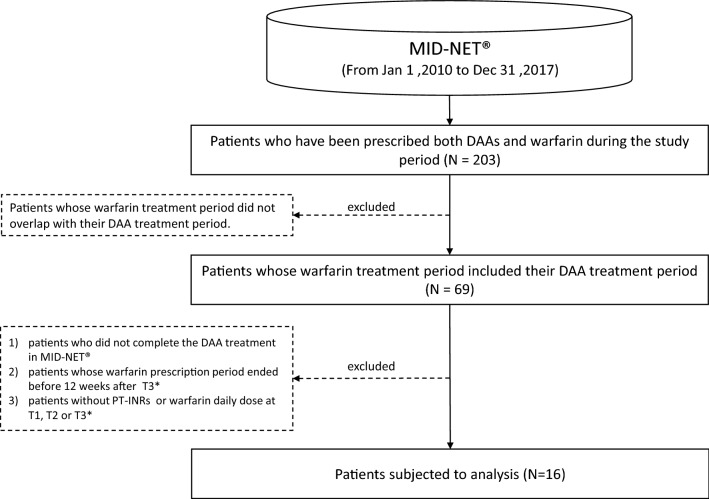
Flow Chart of This Study. *DAAs* direct-acting antivirals against hepatitis C. *T1 date of initiation of the DAA treatment, *T2* date of completion of the DAA treatment, *T3* date when 12 weeks have passed since the completion of the DAA treatment.

The flow chart for patient selection in this study is shown in Fig. 1. Firstly, data for patients who were prescribed both DAAs and warfarin at least one time during the study period were extracted from MID-NET^®^. All DAAs that were marketed in Japan during the study period were investigated in this study, resulting in the following twelve DAAs: daclatasvir hydrochloride, asunaprevir, ombitasvir hydrate/paritaprevir hydrate/ritonavir, sofosbuvir, ledipasvir acetonate/sofosbuvir, elbasvir, grazoprevir hydrate, daclatasvir hydrochloride/asunaprevir/beclabuvir hydrochloride, glecaprevir hydrate/pibrentasvir, telaprevir, vaniprevir, and simeprevir sodium. It should be noted that vaniprevir, telaprevir, ombitasvir hydrate/paritaprevir hydrate/ritonavir, daclatasvir hydrochloride/asunaprevir/beclabuvir hydrochloride, and simeprevir sodium are no longer marketed in Japan [13–17].

Secondly, we identified patients whose warfarin treatment period included also a DAA treatment period. Warfarin and DAA treatment periods were created with a 30-day gap period. Specifically, when the date of the next prescription for a patient was recorded within 30 days after the end date of the previous prescription period, we combined these prescription data as a single treatment period for the patient (see supplement A).

Thirdly, we excluded the following patients: (1) patients who did not complete their DAA treatment. For this criterion, we excluded the patients whose DAA treatment period was more than 15 days shorter than the designated treatment period shown in the package insert, considering some allowances for the designated treatment period for adapting various situations in clinical practice. For instance, as the treatment period with telaprevir was designated as 12 weeks (84 days) in the package insert, patients whose telaprevir treatment period in MID-NET^®^ was less than 69 days were excluded (see supplement B); (2) patients who did not continue receiving warfarin for 12 weeks after completion of the DAA treatment (see supplement C); (3) patients for whom there were no records of PT-INR and/or warfarin daily doses at all the following three time points: at the date of initiation of the DAA treatment (T1), at the date of completion of the DAA treatment (T2), and at 12 weeks after completion of the DAA treatment (T3). When the PT-INRs were not recorded at the exact date of these time points, the most recent PT-INR record within the past 30 days was used for analysis. The warfarin daily dose was calculated by multiplying “units per day” by “unit dosage” at the same date of the PT-INR measurement.

### Outcome Information and Statistical Analysis

To analyze the time trends of the blood coagulability of the patients on warfarin, the mean and standard deviation of the PT-INR, warfarin daily dose (see supplement D), and warfarin sensitivity index (WSI)[18], which was defined as the value obtained by dividing the PT-INR by the warfarin daily dose, were identified for the entire population at the three time points T1, T2, and T3. These time points were selected based on the previous studies [7, 8]. In addition, the percentage along with its mean and standard deviation of the three values (PT-INR, warfarin daily dose, and WSI) at T2 and T3 were calculated against T1 for individual patients.

With regard to the liver function of the patients on warfarin, relevant laboratory test values, such as aspartate aminotransferase [AST], alanine aminotransferase [ALT], alkaline phosphatase [ALP], γ-glutamyl transpeptidase [γ-GTP], index of hepatic fibrosis [FIB-4 index], and platelet count), and hepatitis C viral load, were also investigated.

### Ethical considerations

The application of MID-NET^®^ for this study was approved on June 29, 2018, by the expert committee of MID-NET^®^ [19]. Since this study was conducted as an official activity of the PMDA under the Pharmaceuticals and Medical Devices Agency Law (Article 15–5–(c) and (f))[20], it was not subject to a review by the Institutional Review Boards.

## Results

**Figure 2.**
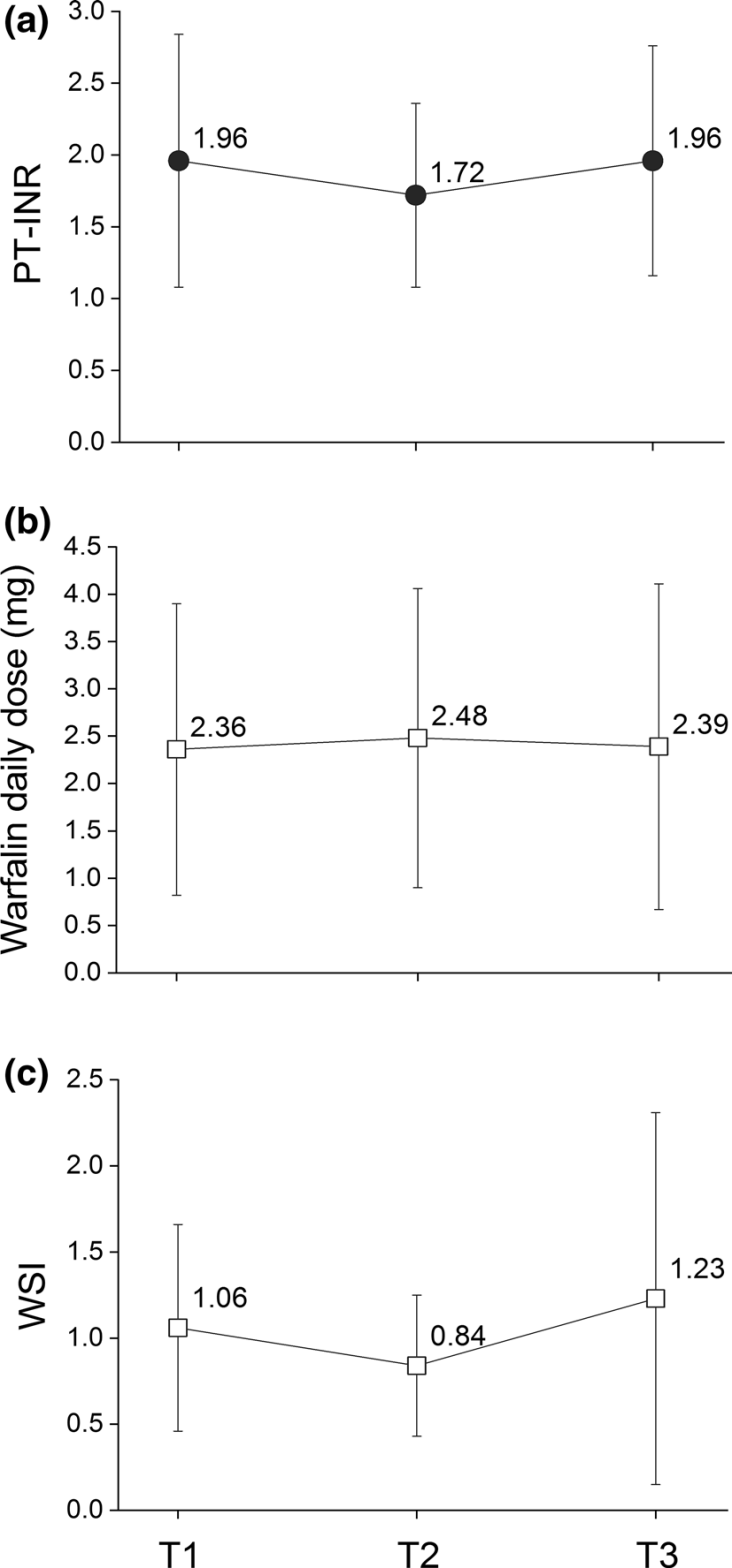
Trends of PT-INR, Warfarin Daily Dose, and WSI at each Time Point in Means and Standard Deviations. Each value is shown as mean value ± standard deviation at the each time point, i.e., T1 (baseline: the date of initiation of the DAA treatment), T2 (completion of the DAA treatment), and T3 (12 weeks after the completion of the DAA treatment). When a test result was not recorded on the exact date of the time point, the most recent test result within the past 30 days was used for analysis. WSI was estimated by dividing the PT-INR by the warfarin daily dose. *DAAs* Direct-acting antivirals against hepatitis C, *WSI* warfarin sensitivity index.

As shown in Fig. 1, a total of 203 patients were identified as a population who received prescriptions of both DAAs and warfarin during the study period. However, after applying all inclusion and exclusion criteria (see “Methods” section), 16 patients with a mean age of 65.8 (SD 11.5) were included for further analysis. The number of patients for each DAA was less than 10 (data not shown due to the MID-NET^®^ publication rule for protecting privacy information). The time trends of PT-INR, warfarin daily dose, and WSI at the three time points are presented in Fig. 2. For the mean value, both PT-INR and WSI decreased at T2 (PT-INR 1.72; WSI 0.84) compared to T1 (PT-INR 1.96; WSI 1.06), while subsequently increasing at T3 (PT-INR 1.96; WSI 1.23). On the other hand, the warfarin daily dose slightly increased at T2 (2.48) compared to T1 (2.36), followed by a decrease at T3 (2.39). Similar trends were observed in the case of the mean of proportion (see Supplementary Table 1 for details).

**Figure 3.**
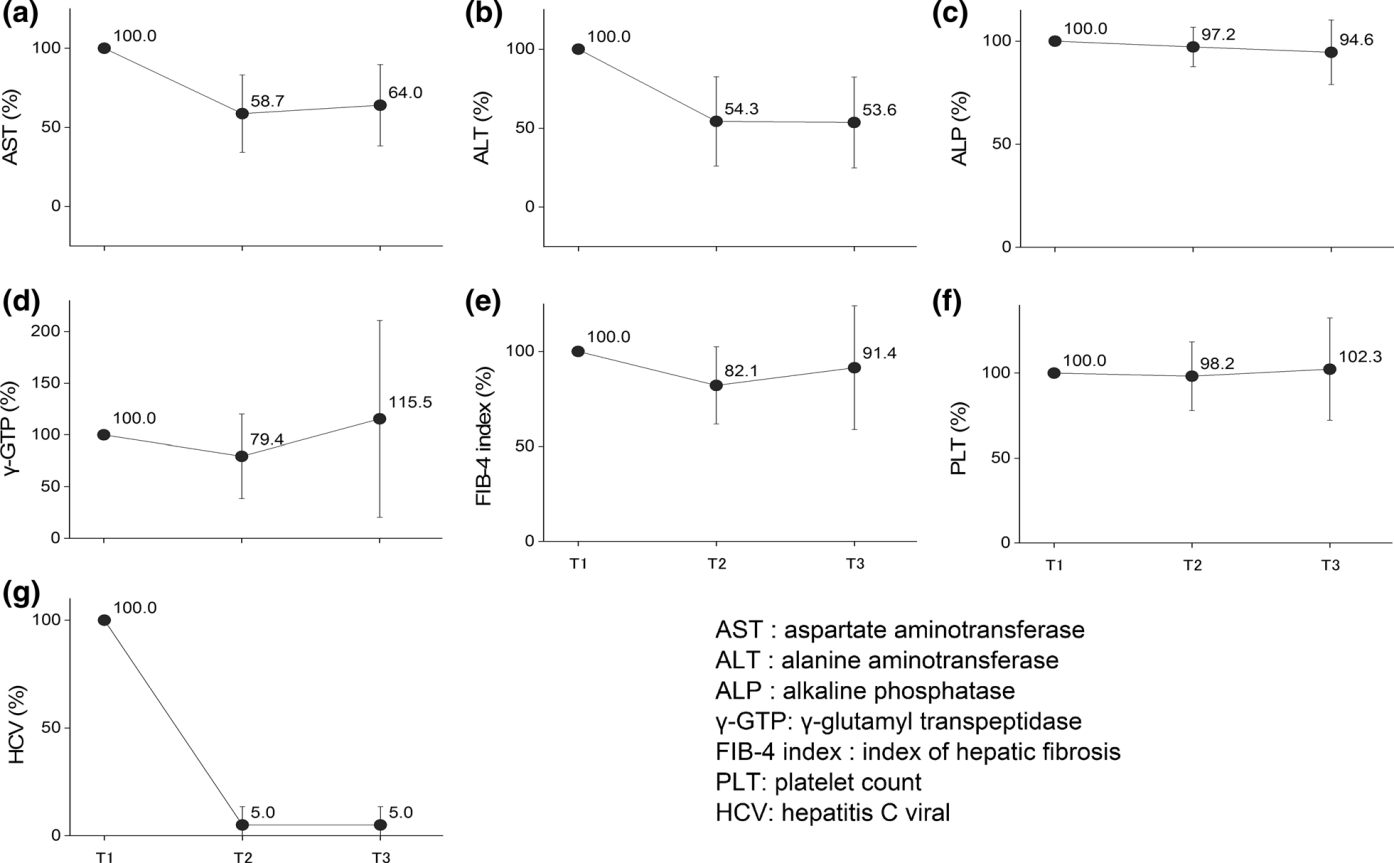
Trends of Liver Function-Related Laboratory Test Results and HCV Viral Load at each Time Point in Mean of Proportion. Each value is shown as mean of proportion ± standard deviation at each time point, i.e., T1 (baseline: date of initiation of the DAA treatment), T2 (completion of the DAA treatment), and T3 (12 weeks after the completion of the DAA treatment). When a test result was not recorded on the exact date of the time point, the most recent test result within the past 30 days was used for analysis.

Furthermore, the time trends of the laboratory test results related to the liver function and hepatitis C viral load are shown in Fig. 3 (see also Supplementary Table 2 for details). In the mean of proportion of the AST and ALT values, an approximate 40–45% decrease was observed at T2, and then these decreased values were maintained at T3 (Fig. 3a and b). The γ-GTP and FIB-4 index decreased by approximately 20% at T2 compared to T1, and subsequently increased with larger variations at T3 (Fig. 3d and e). No marked fluctuations were observed for ALP and platelet count from T1 to T2 and T3 (Fig. 3c and f). In the case of the hepatitis C virus load, a dramatic 95% decrease from T1 was observed at T2, followed by a constant level from T2 to T3 (Fig. 3g).

## Discussion

The effects of DAA prescription on the blood coagulability and liver function in patients on warfarin were descriptively investigated using MID-NET^®^, although the number of the study population was lower than expected. As shown in Fig. 1, this limited number actually resulted from the exclusion of many patients whose DAA prescription period did not overlap with that of warfarin, although 203 patients on prescription of both DAAs and warfarin were identified. This may be related to the characteristics of MID-NET^®^, which is composed of a limited number of university hospitals and regional core hospitals [11]. In these hospitals, warfarin might be less often prescribed than in primary care hospitals or clinics. In addition, the subjects for the analysis further decreased from 69 to 16 patients by the some exclusion criteria including the completion of DAA treatment and having regular laboratory tests at all time points of T1, T2, and T3. Most patients were excluded by the latter criteria despite the allowance period of 2 weeks for laboratory test dates before and after each time point. This would be one of the limitation in using real-world data on which laboratory tests are conducted in case-by-case basis but not regular interval like clinical trials.

When focusing on the results of this study, the changing trend of WSI, which showed a decrease at T2 followed by a re-increase at T3, was similar to that of an American study [8]. The large decrease of both AST and ALT at T2 as well as similar fluctuation trends of γ-GTP and FIB-4 index to that of WSI suggested a possible relation between fluctuations of the liver function with those of blood coagulability. Meanwhile, it seemed unlikely that ribavirin co-administered with DAAs could be a major reason for these effects, owing to the few corresponding patients included in this study. A limited number of population in this study caused difficulty to consider other possibilities such as drug–drug interactions between warfarin and DAAs or ritonavir. With regard to the hepatitis C virus load, a marked decrease at T2 and its lasting effect observed at T3 indicated a valid efficacy of DAAs, although a further study with a longer follow-up period will be needed to assure clinical effectiveness in real-world patients.

The strength of this study was based on the utilization of laboratory test results as outcome, such as PT-INR, warfarin daily dose, WSI, and relevant laboratory test values (AST, ALT, ALP, γ-GTP, FIB-4, platelet count, and hepatitis C viral load) from MID-NET^®^, which was reported to be a reliable database [11]. On the other hand, it was not possible to conduct a detailed analysis, such as that of the blood coagulability for individual DAA regimens, due to the small sample size. The results in this study may also have a limited generalizability to broader patient samples in Japan because of the limited study population as well as the characteristics of the MID-NET^®^-cooperative hospitals as discussed above.

The PMDA conducted a safety assessment of the blood coagulability risk associated with the DAA use based on case reports and literature on subjects with adjustments of the warfarin dose after DAA administration, relevant regulatory safety measures taken by the FDA and EMA [9, 10], and this study results as reference. In February 2020, the PMDA announced a revision of the package insert of DAAs to include a precaution regarding the need for close monitoring during administration of warfarin and other drugs (e.g., antihyperglycemics), whose effectiveness can be affected by the liver function in patients on DAA treatment [21].

## Conclusion

The analysis of MID-NET^®^ data provided useful information on the drug safety assessment in real-world patients, especially in relation to the fluctuation trends of blood coagulability and liver function during DAA treatment in patients on warfarin. It suggests that changes of the liver function may relate to a fluctuation of blood coagulability in patients on both DAA and warfarin. This study greatly contributes to a deeper understanding of the usefulness and limitations of real-world data in MID-NET^®^ for regulatory purposes.

## Supplementary information

Below is the link to the electronic supplementary material.

Supplementary material 1 (PPTX 59 kb)

Supplementary material 2 (DOCX 30 kb)
