# Supplementary material for: Effect of Hepatitis C Drugs on Blood Coagulability in Patients on Warfarin Using the Medical Information Database Network (MID-NET®) in Japan
Source: Ther Innov Regul Sci. 2021 Jan 3;55(3):539–44. doi: 10.1007/s43441-020-00247-8 (PMC8021533; doi:10.1007/s43441-020-00247-8)
Supplement: Supplementary file 1 — Supplementary material 1 (PPTX 59 kb) [file 43441_2020_247_MOESM1_ESM.pptx]

## Slide 1
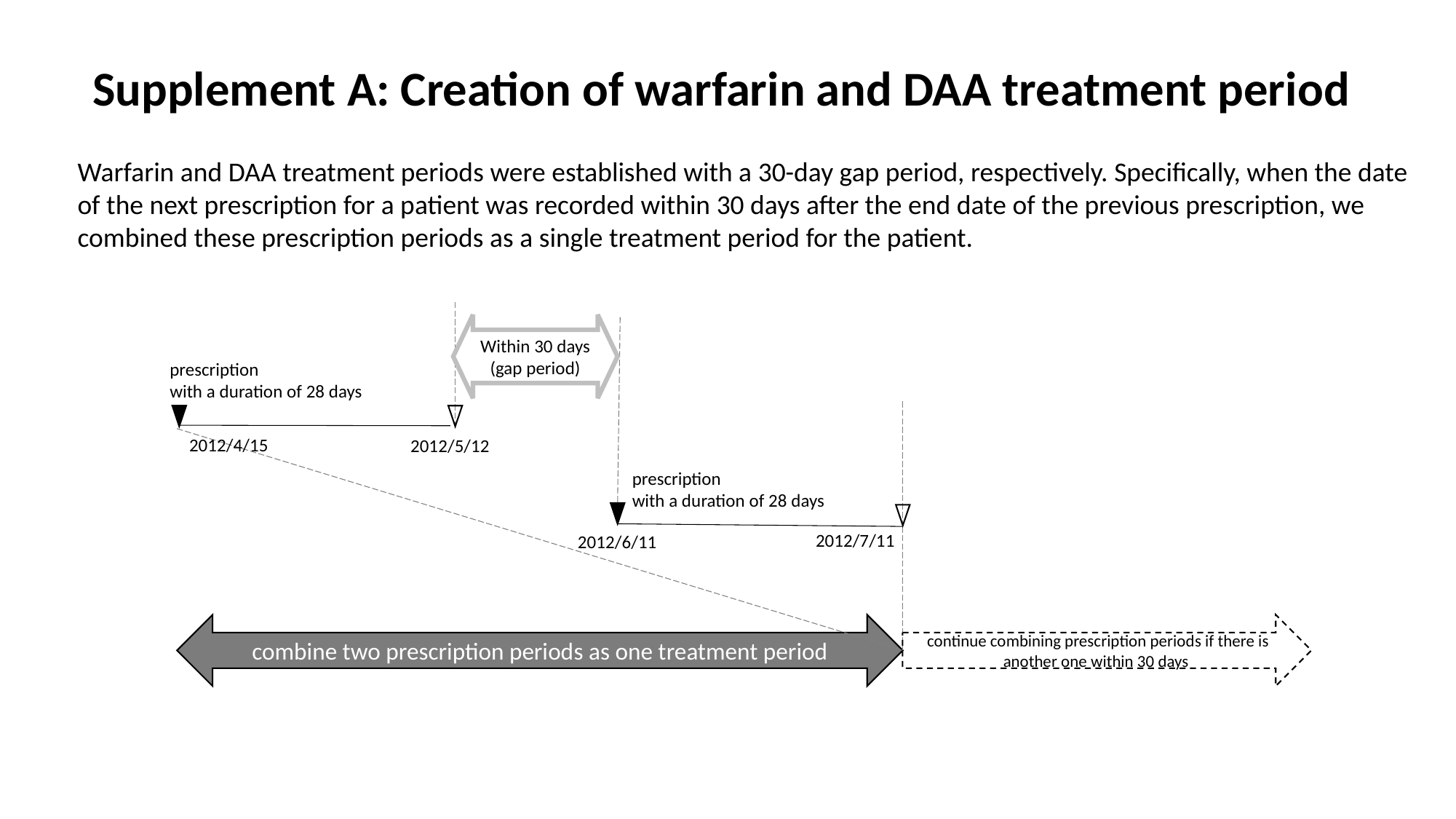

Supplement A: Creation of warfarin and DAA treatment period
Warfarin and DAA treatment periods were established with a 30-day gap period, respectively. Specifically, when the date of the next prescription for a patient was recorded within 30 days after the end date of the previous prescription, we combined these prescription periods as a single treatment period for the patient.
Within 30 days
(gap period)
prescription
with a duration of 28 days
2012/4/15
2012/5/12
prescription
with a duration of 28 days
2012/7/11
2012/6/11
continue combining prescription periods if there is another one within 30 days
combine two prescription periods as one treatment period

## Slide 2
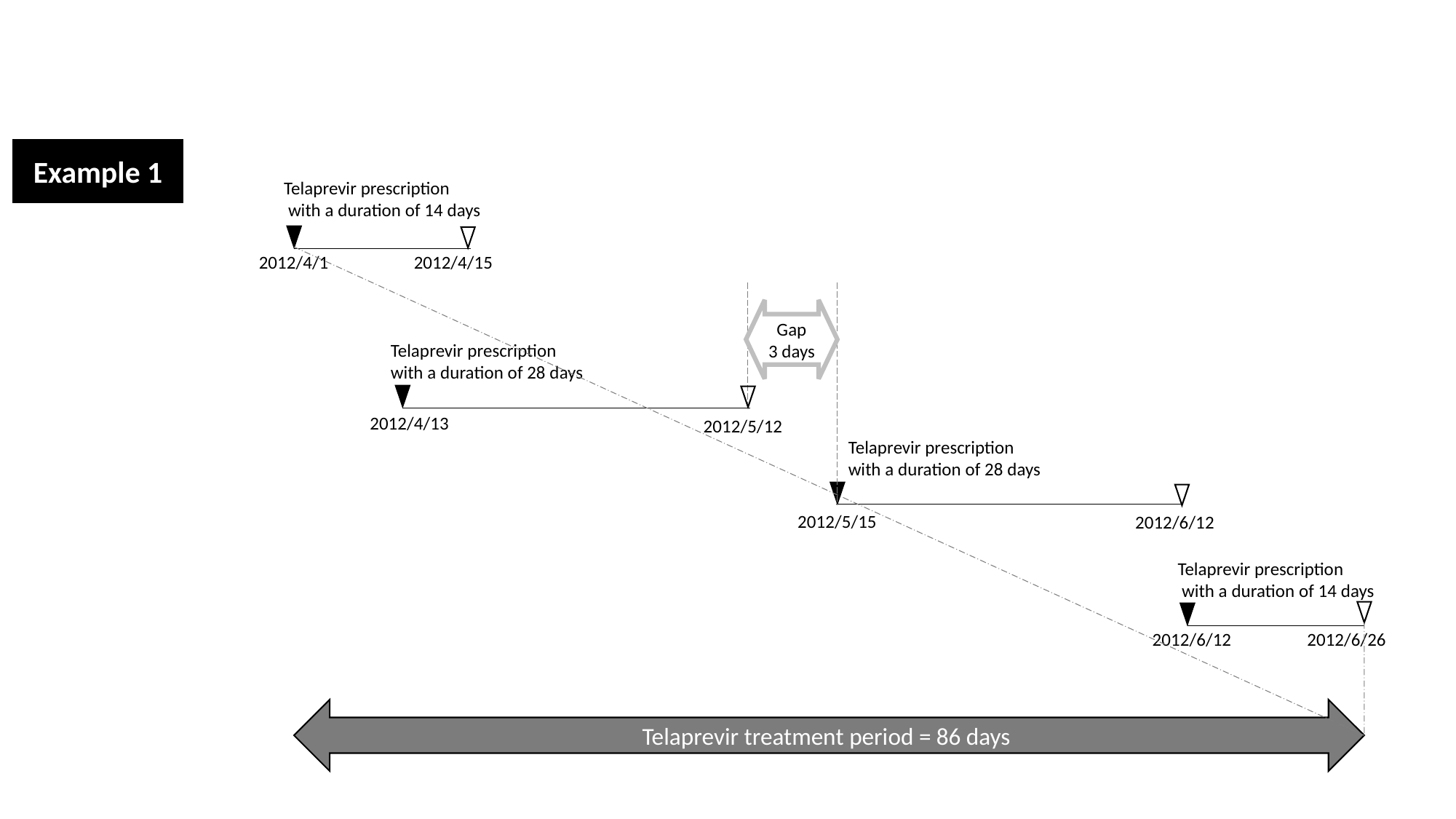

Example 1
Telaprevir prescription
 with a duration of 14 days
2012/4/1
2012/4/15
Gap
3 days
Telaprevir prescription
with a duration of 28 days
2012/4/13
2012/5/12
Telaprevir prescription
with a duration of 28 days
2012/5/15
2012/6/12
Telaprevir prescription
 with a duration of 14 days
2012/6/12
2012/6/26
Telaprevir treatment period = 86 days

## Slide 3
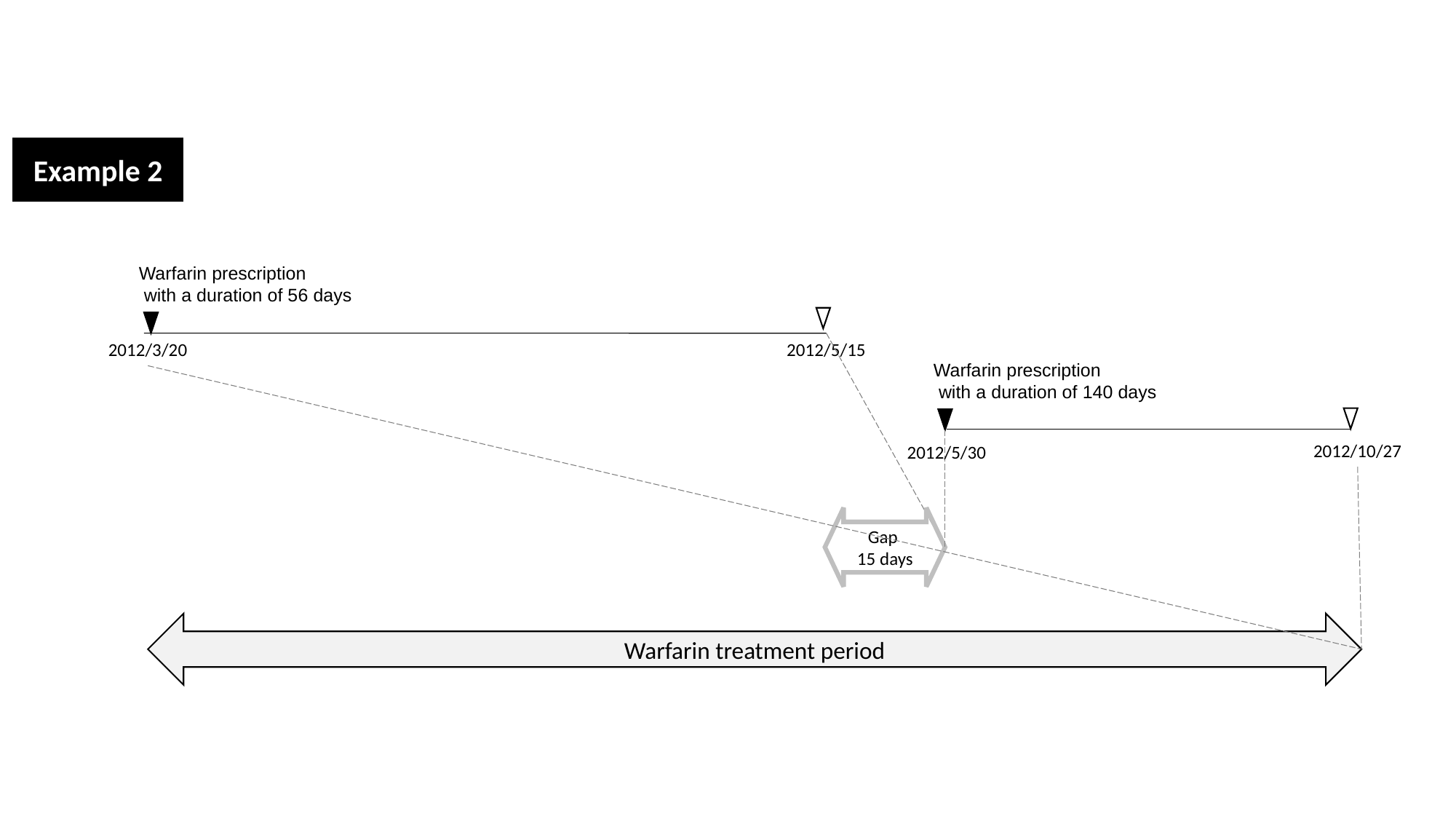

Example 2
Warfarin prescription
 with a duration of 56 days
2012/3/20
2012/5/15
Warfarin prescription
 with a duration of 140 days
2012/10/27
2012/5/30
Gap
15 days
Warfarin treatment period

## Slide 4
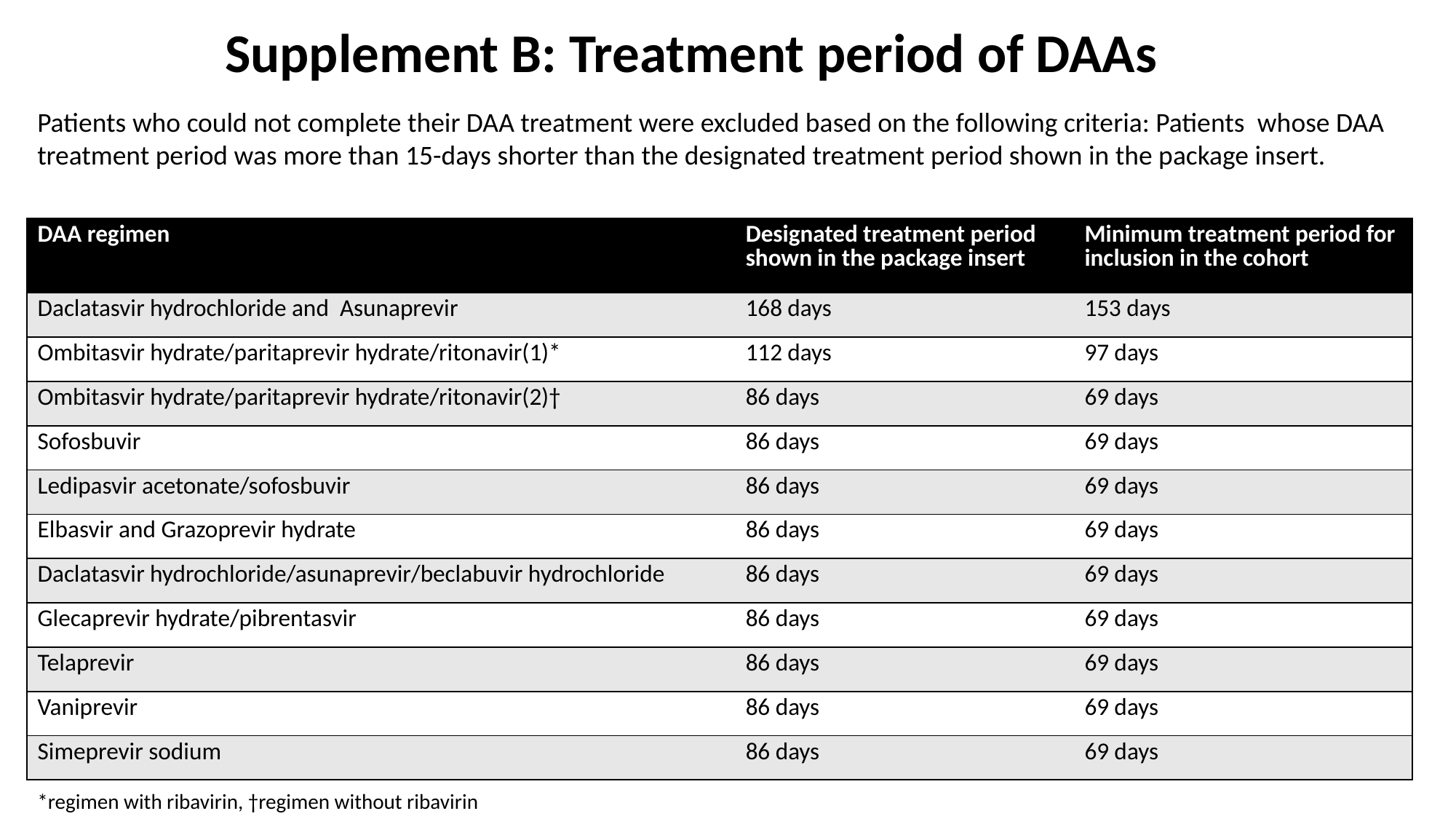

Supplement B: Treatment period of DAAs
Patients who could not complete their DAA treatment were excluded based on the following criteria: Patients  whose DAA treatment period was more than 15-days shorter than the designated treatment period shown in the package insert.
| DAA regimen | Designated treatment period shown in the package insert | Minimum treatment period for inclusion in the cohort |
| --- | --- | --- |
| Daclatasvir hydrochloride and Asunaprevir | 168 days | 153 days |
| Ombitasvir hydrate/paritaprevir hydrate/ritonavir(1)\* | 112 days | 97 days |
| Ombitasvir hydrate/paritaprevir hydrate/ritonavir(2)† | 86 days | 69 days |
| Sofosbuvir | 86 days | 69 days |
| Ledipasvir acetonate/sofosbuvir | 86 days | 69 days |
| Elbasvir and Grazoprevir hydrate | 86 days | 69 days |
| Daclatasvir hydrochloride/asunaprevir/beclabuvir hydrochloride | 86 days | 69 days |
| Glecaprevir hydrate/pibrentasvir | 86 days | 69 days |
| Telaprevir | 86 days | 69 days |
| Vaniprevir | 86 days | 69 days |
| Simeprevir sodium | 86 days | 69 days |
*regimen with ribavirin, †regimen without ribavirin

## Slide 5
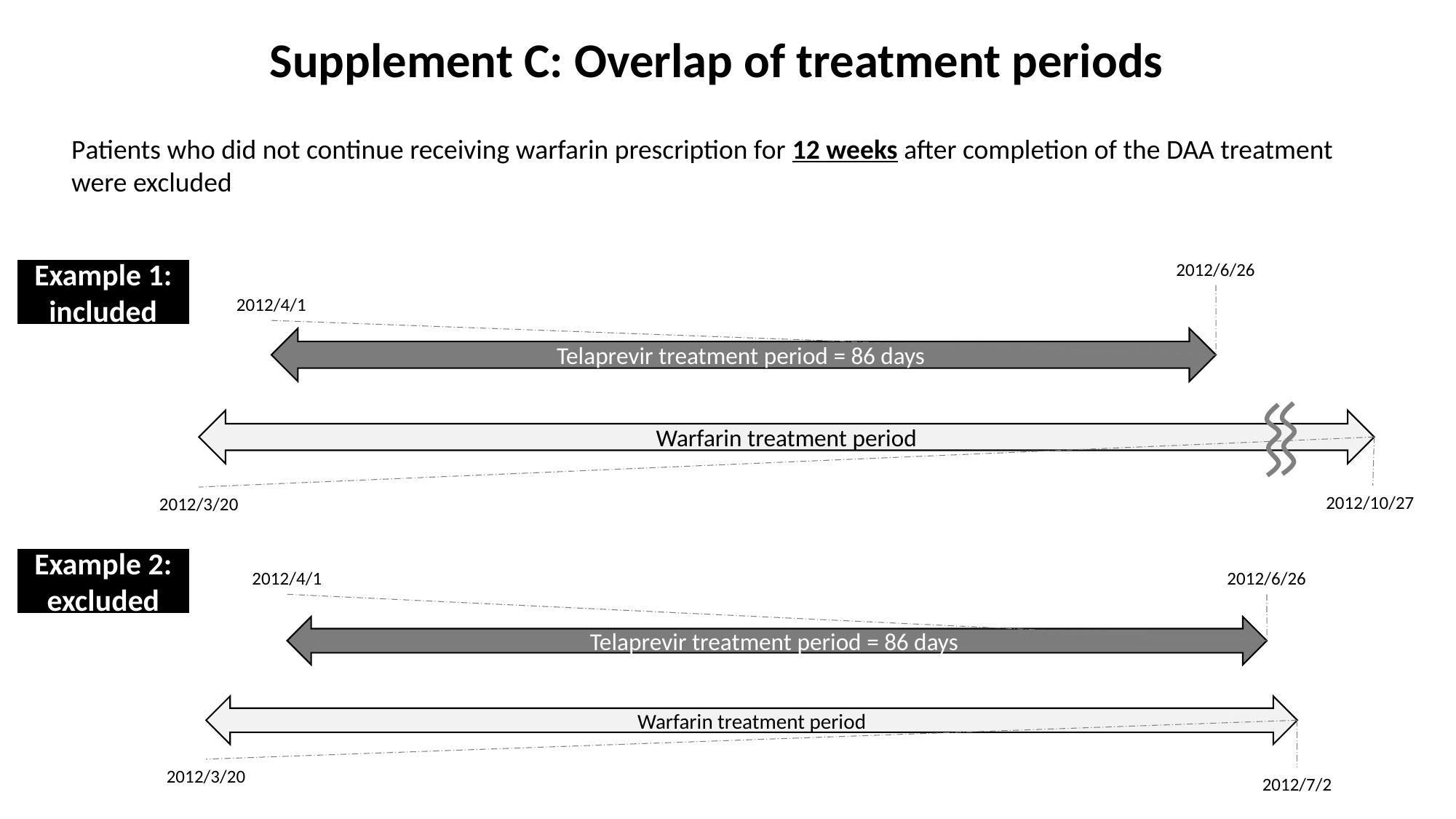

Supplement C: Overlap of treatment periods
Patients who did not continue receiving warfarin prescription for 12 weeks after completion of the DAA treatment were excluded
2012/6/26
Example 1: included
2012/4/1
Telaprevir treatment period = 86 days
Warfarin treatment period
2012/10/27
2012/3/20
Example 2: excluded
2012/6/26
2012/4/1
Telaprevir treatment period = 86 days
Warfarin treatment period
2012/3/20
2012/7/2

## Slide 6
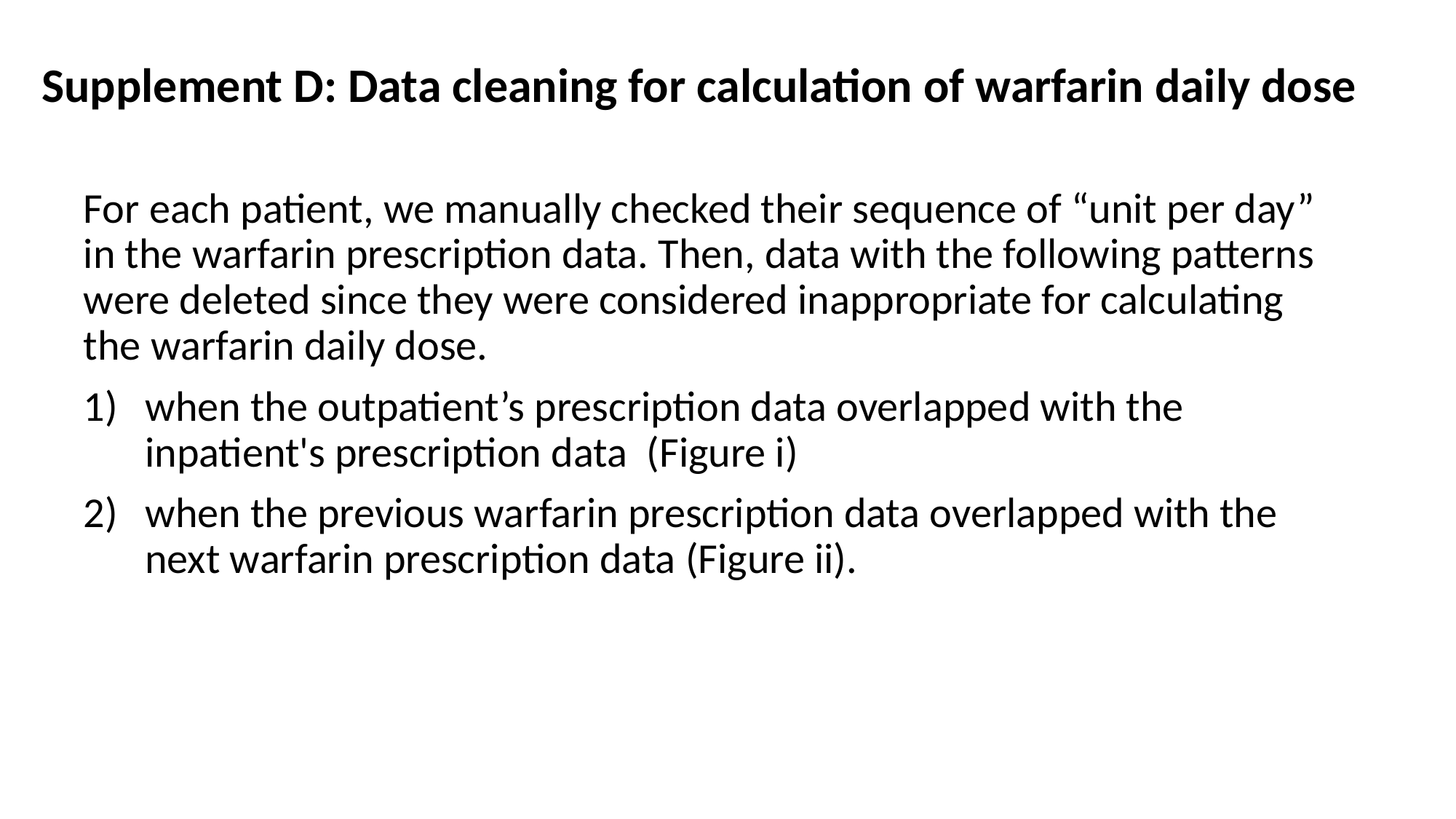

Supplement D: Data cleaning for calculation of warfarin daily dose
For each patient, we manually checked their sequence of “unit per day” in the warfarin prescription data. Then, data with the following patterns were deleted since they were considered inappropriate for calculating the warfarin daily dose.
when the outpatient’s prescription data overlapped with the inpatient's prescription data (Figure i)
when the previous warfarin prescription data overlapped with the next warfarin prescription data (Figure ii).

## Slide 7
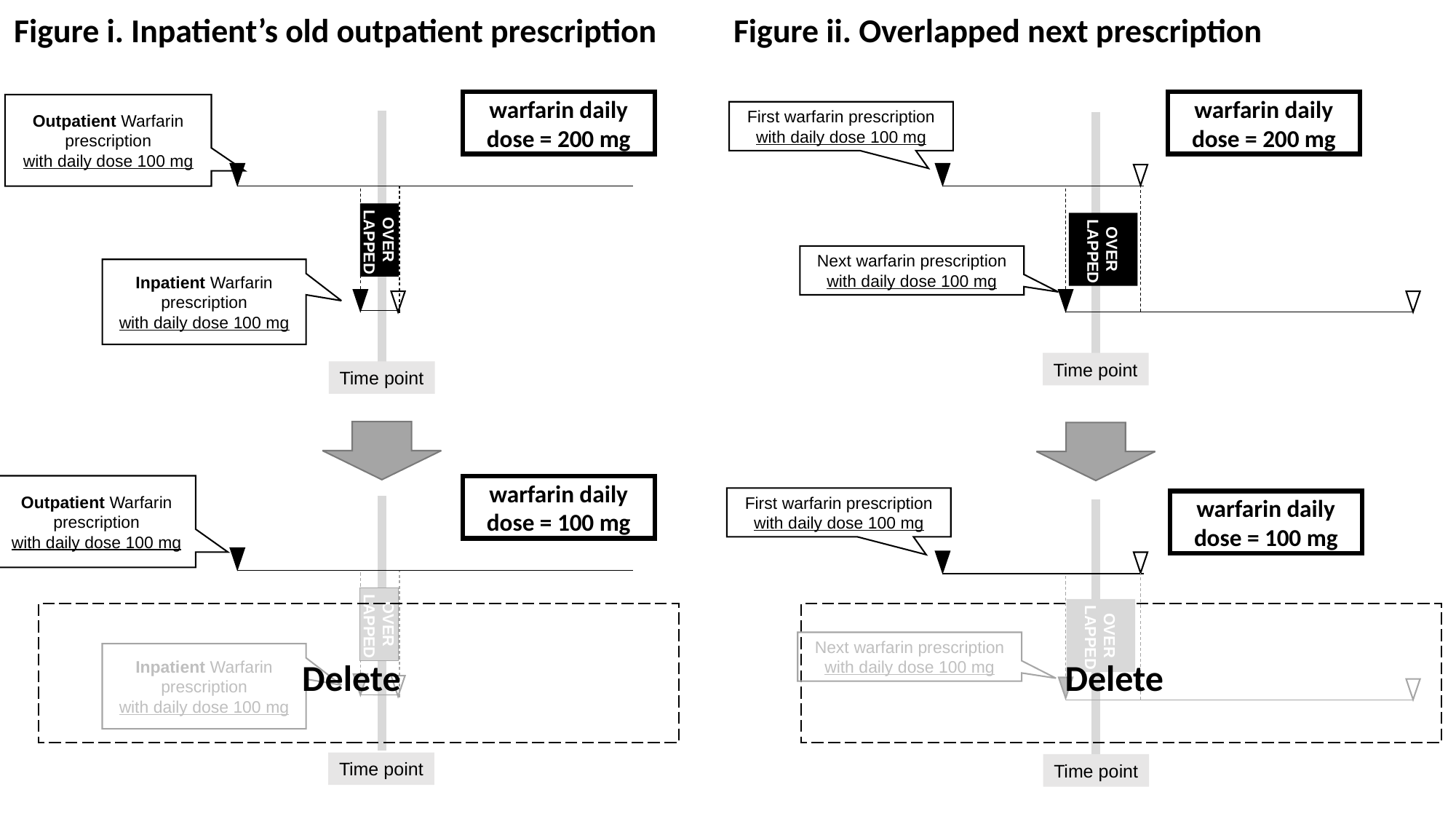

Figure ii. Overlapped next prescription
Figure i. Inpatient’s old outpatient prescription
warfarin daily dose = 200 mg
warfarin daily dose = 200 mg
Outpatient Warfarin prescription
with daily dose 100 mg
First warfarin prescription
with daily dose 100 mg
OVER
 LAPPED
OVER
 LAPPED
Next warfarin prescription
with daily dose 100 mg
Inpatient Warfarin prescription
with daily dose 100 mg
Time point
Time point
Outpatient Warfarin prescription
with daily dose 100 mg
warfarin daily dose = 100 mg
First warfarin prescription
with daily dose 100 mg
warfarin daily dose = 100 mg
OVER
 LAPPED
OVER
 LAPPED
Next warfarin prescription
with daily dose 100 mg
Inpatient Warfarin prescription
with daily dose 100 mg
Delete
Delete
Time point
Time point
