# Supplementary material for: Effect of Hepatitis C Drugs on Blood Coagulability in Patients on Warfarin Using the Medical Information Database Network (MID-NET®) in Japan
Source: Ther Innov Regul Sci. 2021 Jan 3;55(3):539–44. doi: 10.1007/s43441-020-00247-8 (PMC8021533; doi:10.1007/s43441-020-00247-8)
Supplement: Supplementary file 2 — Supplementary material 2 (DOCX 30 kb) [file 43441_2020_247_MOESM2_ESM.docx]

**Supplementary Table 1**

Mean (± standard deviation) of PT-INR, warfarin daily dose, and WSI at each time point (n = 16)

|  | **T1**  Baseline  (date of initiation of the DAA treatment) | **T2**  At completion of the DAA treatment | **T3**  Week 12 after completion of the DAA treatment |
| --- | --- | --- | --- |
| PT-INR |  |  |  |
| Mean_value | 1.96 (± 0.88) | 1.72 (± 0.64) | 1.96 (± 0.80) |
| Mean_proportion | 100% | 96.7% (± 38.7) | 108.2 (± 40.4) |
| Warfarin daily dose  (mg) |  |  |  |
| Mean_value | 2.36 (± 1.54) | 2.48 (± 1.58) | 2.39 (± 1.72) |
| Mean_proportion | 100% | 116.4% (± 40.6) | 108.9% (± 43.6) |
| WSI |  |  |  |
| Mean_value | 1.06 (± 0.60) | 0.84 (± 0.41) | 1.23 (± 1.08) |
| Mean_proportion | 100% | 93.8% (± 54.5) | 115.2% (± 60.1) |

DAAs: Direct acting antivirals against hepatitis C, WSI: warfarin sensitivity index

When a test result was not recorded on that date, the most recent test result within the past 30 days was used. WSI was estimated by dividing the PT-INR by the warfarin daily dose.

**Supplementary Table 2.**

Mean (± standard deviation) of liver-related laboratory test results and HCV viral load at each time point

|  | **T1**  Baseline ^a)^  (Date of initiation of the DAA treatment) | **T2**  At completion of the DAA treatment ^b^ | **T3**  Week 12 after completion of the DAA treatment ^b^ |
| --- | --- | --- | --- |
| AST (N = 15) |  |  |  |
| Mean_value (U/L) | 52.00(± 47.96) | 24.20(± 10.07) | 32.00(± 29.12) |
| Mean_proportion | 100% | 58.7% (± 24.5) | 64.0% (± 25.7) |
| ALT (N = 15) |  |  |  |
| Mean_value (U/L) | 48.13(± 59.52) | 19.33(± 13.49) | 22.40(± 25.57) |
| Mean_proportion | 100% | 54.3% (± 28.3) | 53.6% (± 28.7) |
| ALP (N = 14) |  |  |  |
| Mean_value (U/L) | 435.64(± 324.86) | 414.71(± 291.38) | 433.43(± 401.75) |
| Mean_proportion | 100% | 97.2% (± 9.6) | 94.6% (± 15.6) |
| γ-GTP (N = 15) |  |  |  |
| Mean_value (U/L) | 80.00(± 136.54) | 71.80(± 157.72) | 127.60(± 283.56) |
| Mean_proportion | 100% | 79.4% (± 40.9) | 115.5% (± 95.2) |
| FIB-4 index (N = 12) |  |  |  |
| Mean_value | 5.16(± 8.02) | 3.69(± 4.97) | 4.80(± 8.15) |
| Mean_proportion | 100% | 82.1% (± 20.4) | 91.4% (± 32.6) |
| PLT (N = 15) |  |  |  |
| Mean_value(10^4^/µL) | 17.98(± 10.57) | 16.69(± 7.94) | 16.67(± 6.43) |
| Mean_proportion | 100% | 98.2% (± 20.2) | 102.3% (± 30.2) |
| HCV (N = 10) |  |  |  |
| Mean_value(Log IU/L) | 6.15(± 0.59) | 0.30(± 0.51) | 0.30(± 0.51) |
| Mean_proportion | 100% | 5.0% (± 8.5) | 5.0% (± 8.5) |

AST: aspartate aminotransferase, ALT: alanine aminotransferase, ALP: alkaline phosphatase, γ-GTP: γ-glutamyl transpeptidase, FIB-4 index: index of hepatic fibrosis, PLT: platelet count, HCV: hepatitis C virus

When a test result was not recorded on that date, the most recent test result within the past 30 days was used.
